# Regulation of Wnt Signaling through Ubiquitination and Deubiquitination in Cancers

**DOI:** 10.3390/ijms21113904

**Published:** 2020-05-30

**Authors:** Hong-Beom Park, Ju-Won Kim, Kwang-Hyun Baek

**Affiliations:** Department of Biomedical Science, CHA University, Gyeonggi-Do 13488, Korea; hongbeom5567@naver.com (H.-B.P.); seven1009@naver.com (J.-W.K.)

**Keywords:** cancer, deubiquitinating enzymes, inhibitor, small molecules, ubiquitination, Wnt signaling

## Abstract

The Wnt signaling pathway plays important roles in embryonic development, homeostatic processes, cell differentiation, cell polarity, cell proliferation, and cell migration via the β-catenin binding of Wnt target genes. Dysregulation of Wnt signaling is associated with various diseases such as cancer, aging, Alzheimer’s disease, metabolic disease, and pigmentation disorders. Numerous studies entailing the Wnt signaling pathway have been conducted for various cancers. Diverse signaling factors mediate the up- or down-regulation of Wnt signaling through post-translational modifications (PTMs), and aberrant regulation is associated with several different malignancies in humans. Of the numerous PTMs involved, most Wnt signaling factors are regulated by ubiquitination and deubiquitination. Ubiquitination by E3 ligase attaches ubiquitins to target proteins and usually induces proteasomal degradation of Wnt signaling factors such as β-catenin, Axin, GSK3, and Dvl. Conversely, deubiquitination induced by the deubiquitinating enzymes (DUBs) detaches the ubiquitins and modulates the stability of signaling factors. In this review, we discuss the effects of ubiquitination and deubiquitination on the Wnt signaling pathway, and the inhibitors of DUBs that can be applied for cancer therapeutic strategies.

## 1. Introduction

**Figure 1:**
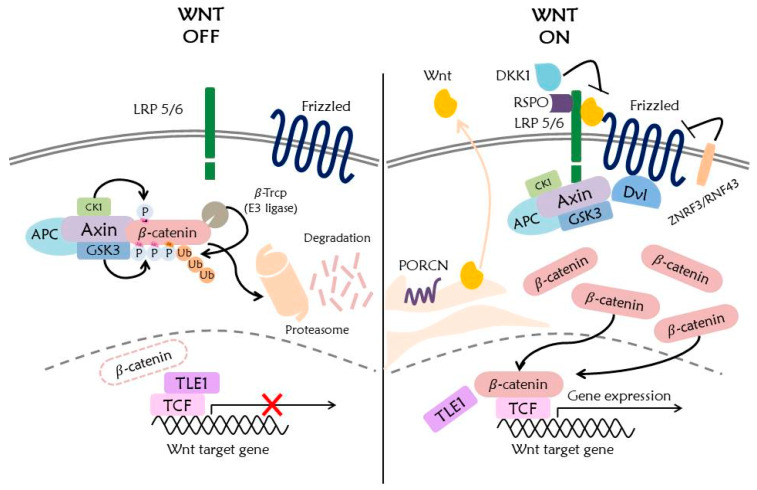
Overview of the Wnt/β-catenin signaling pathway. Without Wnt protein, β-catenin is phosphorylated by a destruction complex including Axin, CK1, GSK3, and APC. Phosphorylated β-catenin is ubiquitinated by β-Tcrp E3 ligase and is degraded by the 26S proteasome. Decreased translocation of β-catenin into the nucleus inhibits the expression of Wnt target genes. With Wnt protein, a destruction complex binds to activated Wnt receptor, resulting in inhibition of phosphorylation and ubiquitination of β-catenin, and subsequent translocation of β-catenin into the nucleus. Binding of β-catenin to TCF induces the expression of the Wnt target genes.

The Wnt signaling pathway mediates numerous cellular functions such as embryonic development, cell proliferation, cell migration, stem cell regulation, and pigment biosynthesis [1,2,3,4]. The Wnt signaling is well known for its embryonic development. Moreover, it also functions for cell differentiation such as melanocytes, which are neural crest-derived cells secreting melanin. The Wnt signaling pathway mediated by Wnt1 and Wnt3a regulates the expression of *MITF* gene, which plays an important role in melanogenesis. Also, it is known that MITF is phosphorylated by ERK, and ubiquitinated and regulated by hUBC9 [5,6]. Recently, it has been found that the Wnt signaling pathway promotes primary ciliogenesis by gene regulation of phosphorylated β-catenin through stimulation of Wnt3a [7]. The Wnt signaling pathway encompasses numerous signaling factors (Figure 1).

In the canonical Wnt signaling pathway, a low-density lipoprotein receptor-related protein 5/6 (LRP5/6), Frizzled receptor (Fz), Disheveled (Dvl), β-catenin, Glycogen Synthase Kinase 3 (GSK3), Casein Kinase 1 (CK1), Axin, Adenomatous polyposis coli (APC), and DNA-bound T cell factor/lymphoid enhancer factor (TCF/LEF) are the known main signaling factors of Wnt signaling [2,8].

Dysregulation or mutation of these signaling factors results in differential gene expression by regulating the amount of β-catenin that induces Wnt signaling target gene expression. Abnormal regulation of Wnt signaling is related to various human pathologies such as cancer, Alzheimer’s disease [9,10], metabolic syndrome [11], and bone diseases [12,13,14]. Researches through the decades reported that the Wnt signaling pathway is related to diverse human cancers, [15,16,17] and have therefore targeted Wnt signaling components for anticancer therapy [18,19,20].

For instance, aggregation and translocation of β-catenin induces Wnt signaling target gene expression, and upregulation of β-catenin is associated with promoting tumor growth of myeloma cells in vitro and prostate cancer cells in vivo [8].

However, in addition to the regulation of Wnt signaling by directly targeting signaling factors, post-translational modifications (PTMs) are also necessary for the Wnt signaling pathway. Most of Wnt signaling proteins undergo one or more PTMs including phosphorylation, glycosylation, palmitoylation, sumoylation, ADP-ribosylation, ubiquitination, and deubiquitination [21].

Among the PTMs, ubiquitination is a well-known mechanism regulating a variety of cellular processes including representative functions, protein degradation, protein–protein interaction, endocytosis, cell cycle progression, and modification of substrate activity [22].

In Wnt signaling, ubiquitination is a major mechanism for regulating the amount of β-catenin. In the absence of Wnt protein, the β-catenin phosphorylated by CK1 and GSK3 is ubiquitinated by binding of β-Trcp, an E3 ligase, and subsequently degraded by the 26S proteasome system [23,24]. In addition to β-catenin, Wnt signaling factors such as Fz, LRP6, Axin, GSK3, APC, Dvl, and TCF/LEF, are ubiquitinated by various types of E3 ligase, and these factors undergo degradation or increasing protein stability depending on the lysine ubiquitinated [8,21].

Moreover, DUBs regulate the stability of Wnt signaling components through deubiquitination, which is an opposite mechanism to ubiquitination. Hence, balanced ubiquitination and deubiquitination is an important process for maintaining stability of the signaling components related to cancer cell growth and metastasis. Anticancer therapy targeting ubiquitination and deubiquitination of the Wnt signaling components, along with the well-known Wnt signaling inhibitors and small molecules, are likely to play an important role in the treatment of various human cancers.

## 2. Components of the Wnt Signaling Pathway and Molecular Mechanisms

Wnt protein is secreted as a cysteine rich protein composed of 350–400 amino acids; in humans, nineteen Wnt genes encode the Wnt proteins [25]. The secreted Wnt proteins bind to different Wnt receptors, resulting in downstream signaling cascades, including canonical β-catenin dependent pathway, noncanonical planar cell polarity (PCP), and Wnt/Ca^2+^ pathway [26].

Before Wnt proteins bind to the Wnt receptors and co-receptors, they undergo PTMs (such as lipid modification [27] and glycosylation [25]) for secretion and activation of the Wnt proteins. Palmitoylation of the Wnt protein is triggered by Porcupine (PORCN), a membrane-bound O-acyltransferase, in the endoplasmic reticulum (ER) [28]. Conversely, the palmitification-protein carboxylesterase (NOTUM) is known to reverse the palmitification of the Wnt ligand [29]. As a result, they are important regulators for the activity of Wnt protein. Palmitoylation of Wnt-5a is an important process for inhibiting the TCF4 transcriptional activity and for stimulating cell migration [30]. Also, palmitoylation of Wnt-3a is essential for the secretory process and β-catenin accumulation in Wnt-3a dependent signaling [31]. Binding of the Wnt protein and cargo protein, that transports the Wnt protein from endoplasmic reticulum (ER) to plasma membrane, requires palmitoylation of Wnt proteins after translation. Wnt proteins also require palmitoylation of a serine residue to bind the representative Wnt signaling receptor, namely Fz receptor [32].

The secreted Wnt proteins interact with other Wnt receptors and co-receptors, including LRP5/6, receptor tyrosine kinase-like orphan receptor (ROR), protein tyrosine kinase 7 (PTK7), receptor tyrosine kinase (RYK), muscle skeletal receptor tyrosine kinase (MUSK), and a proteoglycan family [26]. The expression of Fz and LRP5/6 receptors on the cell surface is regulated by ubiquitination. Fz and LRP5/6 receptors are ubiquitinated by RNF43 and ZNRF3, which are RING domain-containing E3 ligases expressed by Wnt target genes. Also RFN43 inhibits the transcriptional activity of TCF4 by anchoring TCF to the nuclear membrane [33]. Thus, RNF43 and ZNRF3 function as negative regulators of the Wnt signaling pathway [34]. In addition to ubiquitination, other PTMs affecting Wnt receptor regulation are phosphorylation, glycosylation, and palmitoylation [21].

The binding of Wnt protein with Fz and LRP5/6 receptors triggers multimerization of Wnt receptors on the cell surface. It is known that the ligand recognition motif of LRP6 differs, depending on the Wnt protein. Wnt6 binds to LRP6 extracellular domain (E1E2), whereas Wnt3 binds to the third and fourth beta-propeller domains (E3E4) of LRP6. Thus, Wnt3 and Wnt 6 can simultaneously bind to LRP6 [35]. Furthermore, Wnt3 is known to bind to LRP6 competitively with the Dickkopf-related protein 1 (DKK1), which is a Wnt signaling antagonist [36]. Conversely, the R-Spondin (RSPO) family proteins enhance the canonical Wnt signaling pathway to bind to the LRP6 receptor [37], and RSPO2–4 functions as an antagonist of DKK1 by preventing DKK1 binding to LRP6, [38] and also as an antagonist of RNF43/ZNRF3 [34]. Therefore, RNF43, ZNRF3, DKK1, and RSPO proteins are important targets for regulating the Wnt signaling pathway at the receptor level.

Multimerization of Wnt receptors recruits Dvl, which contains three conserved domains and two conserved regions (DIX, PDZ, and DEP domains, and the basic and proline-rich regions). These domains and regions are involved in the interaction of Dvl with other proteins. Dvl functions in both canonical and noncanonical Wnt signaling pathways, depending on which domain of Dvl combines with other proteins. Binding of Axin and GSK3-binding protein (GBP) to DIX and PDZ domains triggers the Wnt/β-catenin dependent signaling cascade that stabilizes β-catenin in the cytosol. However, activation of the PDZ and DEP domains triggers the Wnt/β-catenin independent signaling cascade, including PCP and Ca^2+^ pathway [39,40].

Dvl promotes Wnt signaling by binding to a destruction complex containing Axin, but inhibits Wnt signaling by binding to ZNRF3/RNF43, causing degradation of the Wnt receptor [41]. The Dvl-bound Axin complex triggers phosphorylation of the LRP5/6 at PPPSP motif, recruiting more Axin complex to the cell membrane. Relocation of Axin complex to the cell membrane does not cause the ubiquitin-mediated degradation of β-catenin [8,32,36], and this undegraded β-catenin moves into the nucleus and binds to TCF/LEF to induce the expression of Wnt target genes. A recent study found that β-catenin interacts with the FOXO transcriptional factor instead of TCF/LEF to induce TCF-independent gene expression [42].

In the absence of Wnt protein, there is no activation of Wnt receptors by binding of Wnt protein, and hence no signaling factors, including the destruction complex and Dvl, which are recruited to the cell membrane. The destruction complex subsequently induces phosphorylation and ubiquitination of β-catenin, as follows. First, APC phosphorylated by GSK3, and CK1 recruits β-catenin [43]. β-catenin is transported by APC to Axin after protein phosphatase 2A (PP2A) detaches a phosphate group of APC [8]. The GSK3 and CK1 function as a phosphorylase of β-catenin: CK1 phosphorylates β-catenin at Ser45, after which GSK3 phosphorylates β-catenin at S33, S37, and T41. The phosphorylated β-catenin is recognized by β-Trcp, an E3 ligase [44]. Since the WD40-repeat domain of β-Trcp recognizes phosphorylated β-catenin, any mutation of the β-catenin phosphorylation site or deletion of WD40 repeat domain inhibits ubiquitination of β-catenin by β-Trcp [24]. Ubiquitination of β-catenin is mediated by the SCF (Skp1, Cullin, and F box) E3 ligase complex with β-Trcp [45] and ubiquitinated β-catenin undergoes degradation via the 26S proteasome. However, ubiquitination of β-catenin occurs not only in the cytoplasm but also in the nucleus [8]. The ubiquitination of Wnt signaling factors, including β-catenin, will be discussed in more detail later.

## 3. Wnt Signaling Pathway in Diverse Cancers

The Wnt signaling pathway is related to cell proliferation, epithelial mesenchymal transition (EMT), and cell migration, all closely related to cancer development. Mutation of the Wnt signaling pathway components has been found in several cancer types (Table 1) [46]. The research on Wnt signaling pathway in cancers was initiated when it was first determined that the *Wnt1* gene overexpression induces mouse mammary cancer [47]. In human cancer, *APC* gene mutation was first revealed in colon cancer and colorectal cancer [32]. APC is a component of the destruction complex which promotes ubiquitination of β-catenin and APC functions as a negative regulator of the Wnt signaling pathway and cancer. In addition to colorectal cancer, *APC* mutations are found in familial adenomatous polyposis (FAP), pancreatic, gastric [48], liver, stomach, thyroid, and skin cancers.

Another component of the destruction complex, Axin, also functions as a negative regulator of cancer growth, and two isoforms (Axin1 and Axin2) function equally in the Wnt signaling pathway. Alternations of Axin1 and Axin2 have been detected in diverse human cancers and cancer cell lines [49]. Overexpression of Axin1 inhibits hepatocellular carcinoma (HCC) cell growth [46] and increased expression of *Axin1* by X-ray irradiation inhibits lung cancer cell proliferation and invasion [50]. Also, in melanomas it is known that Axin1 regulates the level of β-catenin that has a synergy effect with a BRAF-V600E inhibitor (PLX4720) [51]. Recently, polymorphisms of the *Axin1* gene were found to be associated with bladder cancer risk [52]. Axin2 has opposing functions in cancers. Deletion or deregulation of *Axin2* is related to adrenal and breast cancers, and *Axin2* gene polymorphism has been identified in lung cancer [53]. However, in vivo studies show that Axin2 functions as a tumor inducer by upregulating transcription factor SNAI1 that induces EMT and metastasis of colorectal cancer [46].

GSK3 and CK1 are important components that induce ubiquitination and degradation of β-catenin. Hence, expression of GSK3 and CK1 has a negative function in the Wnt signaling pathway. Similar to Axin2, the roles of GSK3 in various cancers are controversial. In colon, liver, ovarian, and pancreatic cancers, GSK3 expression is upregulated but in breast and skin cancers, mutation of GSK3 induces tumorigenesis. Also, inhibition of GSK3 promotes EMT and invasiveness of breast cancer [54]. Three isoforms of CK1 function together in the Wnt signaling pathway. Coordinated functions of CK1α, CK1δ, and CK1ε in the Wnt signaling pathway via Dvl were found in colon carcinoma cells. Several cancers show differing mRNA levels of *CK1* isoforms. The mRNA level of *CK1δ* and *CK1ε* is overexpressed in bladder, brain, breast, colorectal, kidney, and lung cancers, whereas overexpressed mRNA level of *CK1α* was found in brain and prostate cancers, and leukemia [55].

At the receptor level, diverse proteins are related to the Wnt signaling cascade. One of the Wnt receptors, LRP5/6 is known as a cancer promoting factor [36,56]. In the cell membrane, regulating RNF43/ZNRF3 and RSPO results in accumulation of LRP5/6 and Fz receptors, making the Wnt receptors more sensitive and promoting the Wnt signaling pathway in spite of low concentration of Wnt proteins [32], whereas phosphorylation of LRP6 by WNT 3 is inhibited by protein kinase N1 (PKN1) and is more sensitive to cell death in melanoma cells [57]. Moreover, LRP5/6 overexpression has been reported in metastatic breast cancer cells. However, binding of LRP5/6 to Fz receptor represses metastasis via the noncanonical Wnt signaling pathway [46]. Considering the above reports, the exact role of LRP5/6 during metastasis remains unclear.

Another Wnt receptor, the Fz receptor is a seven-transmembrane receptor, consisting of 10 factors, Fz-1 to Fz-10 [58]. Each Fz receptor induces the canonical Wnt signaling pathway to bind different Wnt proteins, and overexpression of Fz receptors is mostly associated with different human cancers and poor prognosis in cancer. Reports indicate that expression of Fz receptors regulates cell adhesion, EMT, cell migration, cell proliferation, and drug resistance in several cancers through stabilizing β-catenin, several proteins, and signaling pathways such as PKC, STAT3, NF-κB, and CaMKII-TAK1-NLK signaling [59]. Expression of Fz-7 is upregulated in esophageal squamous cell carcinoma (ESCC) patients and overexpression of Fz-7 induces activity of β-catenin, epithelial markers, and mesenchymal markers related to EMT [60]. It is also reported that Fz receptors act as tumor suppressors. Fz-2 represses cell migration and growth in salivary adenoid cystic carcinomas, whereas Fz-6 inhibits migration and cell proliferation in gastric cancer. Also, a low expression of Fz-8 is known to inhibit the Wnt/Ca^2+^ signaling pathway, and consequently increase tumorigenicity [59].

A recent study revealed that the retinoid-related orphan receptor α (RORα) inhibits EMT and invasion in gastric cancer. Overexpression of RORα downregulates Wnt1, β-catenin, Axin, TCF, c-Myc, and c-Jun in MGC803 cells. Thus, it functions as a negative regulator of the Wnt signaling pathway [61].

Dvl is involved in both canonical and noncanonical pathways by transducing signals induced by Wnt proteins. Dvl functions as a positive regulator of Wnt signaling by increasing the stability of β-catenin. Hence, several cancers show an overexpression of Dvl [62]. It has recently been demonstrated that abnormal spindle-like microcephaly associated (ASPM) increases the stability of β-catenin through binding with Dvl in prostate cancer [63].

RNF43/ZNRF3 is an important regulator for Wnt signaling, which controls the stability of Fz receptors through ubiquitination, and mutation of *RNF43/ZNRF3* is known to induce canonical Wnt signaling by improperly maintaining stability of β-catenin. *RNF43/ZRNF3* mutation is found in several cancers, including pancreatic, adrenal, and stomach cancers. Most mutations of *RNF43/ZRNF3* inactivate mutations such as missense and truncating mutations [32]. It has recently been reported that a mutation lacking the RING domain showed no downregulation of the Fz receptor. However, the G659fs mutation, a common mutation of RNF43, functions like the normal RNF43, by inhibiting the Fz receptor activity and is inhibited by RSPO. Taken together, these results indicate that the C-terminal RNF43 mutation that occurs with the β-catenin enhancing mutations induces Wnt signaling [64]. Activation of the Wnt signaling pathway occurs in colon cancer cells with both RNF43 and BRAF-V600E mutations [34].

*CTNNB1* gene encodes β-catenin, an important regulator in the Wnt signaling pathway. Mutation of *CTNNB1* exon 3 triggers altered β-catenin activity causing tumorigenesis in diverse cancers such as adrenal, colorectal, liver, skin, and ovarian cancers [32]. It is known that *CTNNB1* mutation mainly occurs at the N-terminal domain, which is a phosphorylation site. It allows β-catenin to avoid the phosphorylation process via the destruction complex, thereby stabilizing the activity of β-catenin [65]. A recent proteogenomic analysis confirmed that protein levels of APC and Axin are upregulated in *CTNNB1* mutated tumors [66].

The TCF/LEF family regulates the expression of Wnt target genes. The undegraded β-catenin in the cytoplasm moves into the nucleus and binds to TCF/LEF, which is inhibited by binding with other cofactors in the absence of β-catenin. The TCF/LEF family exists in several isoforms by alternative splicing; the isoforms differ in functions, depending on which domain is included by alternative splicing. TCF3 and TCF4 are commonly known to inhibit Wnt target gene, but TCF1 and LEF1 are known to activate Wnt target genes [67]. There are several isoforms of TCF4: TCF4K is known as a tumor growth promoting factor, and TCF4J is known as the tumor growth suppressor [46].

β-Trcp has an important role as an E3 ligase that functions on ubiquitination of β-catenin. Hence, it is a negative regulator in transcription of Wnt target genes, and mutation of β-Trcp induces expression of Wnt target genes interrupting ubiquitination of β-catenin. Also, β-Trcp functions in ubiquitination of several proteins as well as β-catenin. β-Trcp regulates the Discs large homolog 5 (Dlg5) that inhibit HCC cell proliferation, [68] and AE Binding Protein 2 (AEBP2) that induces ovarian cancer cells proliferation and cisplatin resistance [69]. In addition, β-Trcp regulates stability of the yes-associated protein 1 (YAP1) known as an oncogene in pancreatic cancer, [70] and of Mxi1 that represses transcriptional activity of c-Myc in lung cancer through ubiquitination and proteasomal degradation [71].

In MITF mediated by the Wnt signaling pathway it was known that the protein level in amelanotic melanoma is decreased by the anticancer effect of perphenazine and prochlorperazine [72]. In addition to the Wnt signaling pathway factors discussed earlier, other factors that inhibit or promote Wnt signaling pathway (such as RSPO, DKK1, PORCN) are also associated with a number of human cancers (Table 1).

## 4. Ubiquitination System

The ubiquitin system has numerous important roles in regulating cell functions including cell cycle, DNA repair, and signal transduction. Ubiquitination is a process where ubiquitin conjugates to a target protein through a cascade complex, accomplished via E1 (ubiquitin-activating enzyme), E2 (ubiquitin-conjugating enzyme), and E3 ligase (ubiquitin ligase) [87,88]. E1 activates ubiquitin and forms ubiquitin adenylate by using ATP. This activated ubiquitin is transmitted to E2. Finally, the E3 ligase transfers ubiquitin to a specific substrate [87,89]. This transmission generates an isopeptide bond between ubiquitin and the lysine residue of the substrate. Sometimes, ubiquitin combines with non-lysine residues of the substrate, such as serine, threonine, and cysteine. The combined ubiquitin forms a poly-ubiquitin with additional ubiquitins [90]. This combined site is the 7 lysine residue (K6, K11, K27, K29, K33, K48, and K63) or methionine 1 (Met1) site of ubiquitin [91,92,93].

**Figure 2:**
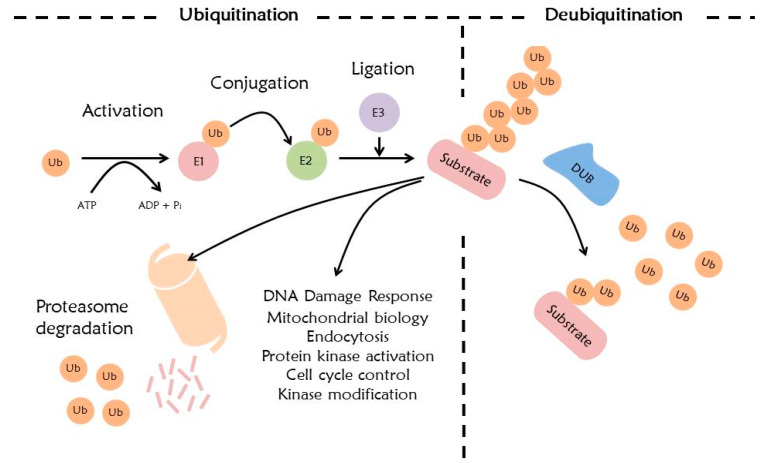
Ubiquitination and deubiquitination system. Ubiquitination proceeds in three steps: activation, conjugation, and ligation through E1, E2, and E3 enzymes. Ubiquitinated substrate is degraded by the 26S proteasome or functions in various other cellular pathways. Deubiquitination is the opposite mechanism of ubiquitination, removing the ubiquitin of the substrate by DUBs.

Eukaryotic cells operate two major protein degradation systems, namely ubiquitin proteasome system (UPS) and autophagy. The UPS is a specific protein degradation system that is activated by binding ubiquitin to its substrates [94]. The ubiquitin-tagged protein can be degraded by 26S proteasome [22]. Especially, ubiquitination at ubiquitin K11 and K48 is recognized as a protein degradation signal by the proteasome. Although the 26S proteasome recognizes a protease signal base on polyubiquitination, it affects the UPS selectivity and specificity by various E3 ligases [95]. The ubiquitin-tagged protein which enters the 26S proteasome is divided into ubiquitin and a protein, and the divided protein is degraded into peptide forms by the proteasome. This divided ubiquitin can further be recycled (Figure 2) [96]. Ubiquitination at ubiquitin K63 is involved in autophagic degradation and DNA damage response [90,95]. Ubiquitination at ubiquitin K6 is involved in DNA repair and mitochondrial homeostasis, ubiquitin K27 is involved in nonproteolytic signaling pathways generating molecules that interact with the ubiquitin binding domains (UBDs) of other proteins, ubiquitin K29 and K33 are involved in kinase modification, and ubiquitin Met1 is involved in NF-kB signaling [90,95,97].

Activation of ubiquitin is regulated by an E1 ligase in the presence of ATP [98]. The intermediate product of this process is a ubiquitin AMP, and the final product is a ubiquitin-E1 ligase thioester bond. The thioester bond is recognized by multiple E2 ligases, and this E2 ligase transfers ubiquitin to another thioester linkage [99]. Hence, the E2 ligase is also called the ubiquitin-conjugating enzyme. The E2 ligase further interacts with E3 ligase, which finally selects the specific target proteins [100]. E3 ligase mainly has an E6-AP carboxyl terminus (HECT) domain; this HECT domain protein forms a thioester bond before transmission of the ubiquitin to a substrate [101]. Proteins which do not have the HECT domain have the RING domain that includes cysteine and histidine amino acids. Unlike the HECT domain, ubiquitination dependent on RING E3 ligase do not have the thioester bond, and the RING E3 ligase directly transfers ubiquitin to its target proteins [102]. RING-in-between-RING (RBR) E3 catalyzes ubiquitin conjugation by directly forming a thioester intermediate with a cysteine of RING2 domain like the RING/HECT mechanism. Various RBRs (Parkin, HHARI, TRIAD1, HOIP and HOIL-1L) are involved in important cellular functions: transcription, translation, regulation of post-translational modifications, and cellular signaling. The misregulation of RBR proteins can affect various diseases including Parkinson disease. Thus, the plausible pharmacological interventions in utilizing these proteins towards therapeutic strategies is possible [103]. The U-BOX, which is composed of approximately seventy amino acids, has a similar tertiary structure to the RING domain. The difference between U-BOX and RING domains is that U-BOX does not have zinc-chelating cysteine and histidine residues [104]. PHD-finger genes have the homeodomain at the C-terminal region. PHD-finger domain has a cysteine-rich region [105].

Diversity of ubiquitination types includes monoubiquitination, multiubiquitination, and polyubiquitination, each having different cellular functions [106]. Monoubiquitination is involved in DNA repair, gene expression, and receptor endocytosis. Monoubiquitination of multiple parts or multiubiquitination is important for receptor endocytosis [107]. Polyubiquitination of Ub-K48 targets a protein that needs to be degraded. Similarly, polyubiquitination of K11 results in proteasomal degradation [108]. The K63-linked ubiquitin regulates the activation of kinase signaling, resistance of DNA damage, and endocytosis [109].

## 5. Deubiquitination System

DUBs remove ubiquitin or ubiquitin-like proteins from target proteins [110]. This process is called deubiquitination. This is a reversible process, and has important roles in the ubiquitin signaling pathway [111]. DUBs mainly have two roles: they are involved in recycling and conversion of ubiquitin which is a result of ubiquitination, and, they rearrange the ubiquitin-linked protein (Figure 2) [112].

**Figure 3:**
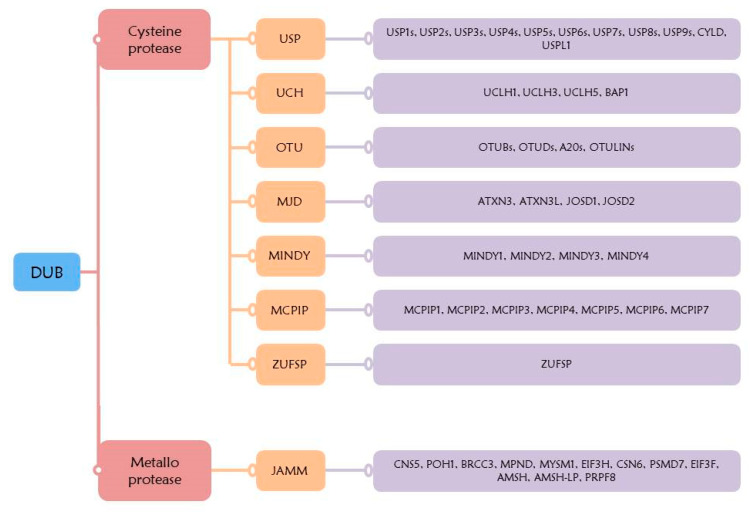
Overview of the DUB family. The DUB family is divided into two subfamilies, based on the enzymatic cleavage mechanism. The cysteine protease family consists of USP, UCH, OTU, MJD, MINDY, MCPIP, and ZUFSP. Metalloprotease family consists of JAMM.

In humans, there are eight subfamilies of DUBs, containing almost one hundred different DUBs. These subclasses are divided into two types based on their enzymatic cleavage mechanism, namely cysteine protease and metalloprotease. The, cysteine protease subclass includes seven families, comprising ubiquitin-specific proteases (USP), ubiquitin C-terminal hydrolases (UCH), Otubain domain ubiquitin-binding proteins (OTU), Machado–Joseph disease protein domain proteases (MJD), MIU-containing novel DUB family (MINDY), monocyte chemotactic protein-induced proteins (MCPIP) families, and ZUFSP family. The subclass metalloprotease includes the Jab1/MPN domain-associated metalloisopeptidase (JAMM) family (Figure 3) [113,114,115]. Mechanism of the cysteine protease DUB is achieved through a nucleophilic attack on the isopeptide linkage of lysine residues ubiquitinated by catalytic cysteine. This process is facilitated by the side chain of histidine, which reduces the pKa of cysteine [116,117]. Mechanism of the metalloprotease (JAMM) is at the catalytic site, and is regulated by zinc ions which are coordinated by histidine and aspartic acid [118].

### 5.1. USP

The USP family has the most number of DUBs; namely, USP1, USP2, USP3, USP4, USP5, USP6, USP7, USP8, USP9X, USP9Y, USP10, USP11, USP12, USP13, USP14, USP15, USP16, USP17L1, USP17L2, USP18, USP19, USP20, USP21, USP22, USP24, USP25, USP26, USP27X, USP28, USP29, USP30, USP31, USP32, USP33, USP34, USP35, USP36, USP37, USP38, USP39, USP40, USP41, USP42, USP43, USP44, USP45, USP46, USP47, USP48, USP49, USP50, USP51, USP52, USP53, USP54, CYLD, and USPL1 [119]. The USP family has a USP domain comprising three subdomains of right-handed palm, thumb, and finger. The cysteine in the active site, localized in the three subdomains, is involved in the interaction with ubiquitin [120]. CYLD is the only USP having the B-box domain [121]. Additional domain and terminal extensions also appear in other USPs, which are important for DUB specificity. For example, USP44, USP45, USP49, and USP51 have zinc finger USP domains [122]. USP25 and USP37 have ubiquitin interaction motifs [123]. USP5 and USP13 have ubiquitin-associated domains, and USP4, USP11, USP15, USP20, USP33, and USP48 have a domain in USP (DUSP) [124]. Moreover, USP4, USP9X, USP9Y, USP11, USP15, USP24, USP32, USP34, USP47, and USP48 have ubiquitin-like domains [125].

### 5.2. UCH

The UCH family has four DUBs in humans; namely, UCHL-1, UCHL-3, UCHL-5, and BAP1. UCH has a short catalytic domain, consisting of 200–300 amino acids [124]. The smaller UCH DUBs (UCH-L1 and UCH-L3) remove small groups from the C-terminus of ubiquitin, while the larger UCH DUBs (UCH37 and BAP1) remove polyubiquitin chains [126]. Interestingly, UCHL-1 has deubiquitination activity as well as dimerization-dependent ubiquitin ligase activity, and also functions as a monoubiquitin stabilizer [127]. UCH-L3 binds to K48-linked polyubiquitin to protect against degradation and inhibit hydrolytic enzyme activity [128]. UCHL-5 is responsible for deubiquitination activity in the 19S proteasome regulatory complex [129]. BAP1 encodes a DUB, which functions to remove monoubiquitin from K119 of histone 2A [130]. BAP1 has the C-terminal extension of about 500 amino acids. The C-terminal extension of BAP1 contains a nuclear position signal and helps to interact with the N-terminal RING finger (ubiquitin ligase) of BRCA1 [131].

### 5.3. OTU

In humans, the OTU family contains eighteen DUBs; namely, OTUB1, OTUB2, OTUD1, OTUD3, OTUD4, OTUD5, OTUD6A, OTUD6B, ALG13, YOD1, HIN1L, A20, OTUD7A, OTUD7B, TRABID, VCPIP, OTULIN, and FAM105A [132]. The OTU family has putative catalytic cysteine and histidine residues, and affects cancer, immunity, and viral immunity. In general, OTU is an isopeptide that catalyzes the breakdown of proteins present between the ε-side chain of lysine and the carboxyl group at the C-terminus [116]. Most OTUs have an OTU catalytic domain, a ubiquitin interaction domain, and a ZnF (Zinc finger) domain. Based on the structure and domain of the protein, phylogenetic analysis delineates four subfamilies of OTU-type DUB: the Otubain subfamily (OTUB1 and OTUB2), the OTUD subfamily (OTUD1, OTUD3, OTUD4, OTUD5, OTUD6A, OTUD6B, OTUD7A, OTUD7B ALG13, YOD1, HIN1L), the A20-like OTUs subfamily (A20, Trabid, VCPIP1), and the OTULIN subfamily (OTULIN, FAM105A) [113,133,134]. Although the structure and sequence of OTUs is different compared to other DUBs, the arrangement of catalytic cysteine and histidine residues remains similar [135].

### 5.4. MDJ

The MDJ class is named after the neurodegenerative Machado–Joseph disease [136]. The MDJ family includes four DUBs; namely, JOSD1, JOSD2, ATXN3-like, and ATXN3 [137]. ATXN3 has the ability to remove the K48- and K63-linked polyubiquitin chains and affect protein folding and stability by removing polyubiquitin [138]. JOSD1, JOSD2, and ATXN3L have a catalytic domain composed of one cysteine and two histidine residues [134].

### 5.5. MINDY

The MINDY family is a recently identified family, and the MINDY domain is a ubiquitin binding domain specific to K48. The MINDY family has four DUBs; namely, MINDY1, MINDY2, MINDY3, and MINDY4 [139]. Full-length MINDY-1 has a high preference for long K48, which removes ubiquitin at the terminal domain [114]. Structurally, the MINDY domain has the same folding as other cysteine proteases [140].

### 5.6. MCPIP

The MCPIP family has seven DUBs; namely, MCPIP1, MCPIP2, MCPIP3, MCPIP4, MCPIP5, MCPIP6, MCPIP7 [113]. MCPIP family members contain two domains which are the CCCH zinc finger and NYN nuclease domains [141]. MCPIP1 interacts with the ubiquitinated protein through the N-terminal ubiquitin-related domain. Other domains of MCPIP1 include the N-terminal conserved region, the CCCH-type zinc finger domain, and the C-terminal proline-rich domain. The catalytic domain consists of cysteine and aspartic acid, but there is no histidine in the catalyst core [142].

### 5.7. ZUFSP

The recently discovered ZUFSP family belongs to the cysteine protease subclass. ZUFSP removes K63-linked polyubiquitin and requires two domains for the process: the ZHA (ZUFSP helical arm), and the atypical UBZ. ZHA binds to distal ubiquitin, where R248, E252, Q259, and Q264 of ZHA, and K63, I44, and A46 of ubiquitin, affect the binding of distal ubiquitin to ZHA. ZUFSP’s atypical UBZ is ZNF4. ZUFSP affects DNA replication and repair by removing the K63-linked ubiquitin [115,143,144].

### 5.8. JAMM

Unlike other DUBs, the JAMM family is a metalloprotease [118]. The JAMM family includes twelve DUBs; namely, CNS5, POH1, BRCC3, MPND, MYSM1, EIF3H, CSN6, PSMD7, EIF3F, AMSH, AMSH-LP, and PRPF8 [134]. Members of the AMSH family of JAMM are involved in the removal of the K63-linked polyubiquitin chain [145]. JAMM protease, which has no AMSH-specific insertion, does not show specificity for the K63-linked polyubiquitin [110]. Seven of the twelve JAMM proteins have activity against ubiquitin or ubiquitin-like proteins, whereas the rest are catalytically inactive [134].

## 6. Ubiquitination in Wnt Signaling Pathway

### 6.1. β-Catenin

In the Wnt signaling pathway, UPS is an important mechanism that regulates the stability of β-catenin, a major signaling factor inducing expression of Wnt target genes. β-Trcp, a ring-type E3 ligase, is known as a representative E3 ligase of β-catenin [24]. The SCF complex (including β-Trcp) attaches the polyubiquitin chain to K19 and K49 of β-catenin by binding the substrate recognition motif of β-catenin [146]. Siah-1, another E3 ligase of β-catenin, induces ubiquitination K666 and K671 of β-catenin, along with the E2 enzyme UbcH5a [147]. The ubiquitination of cytoplasmic β-catenin by Siah-1 is caused by the highly expressed Siah-1 on activation of p53 and, unlike β-Trcp, it induces ubiquitination and degradation regardless of the phosphorylation state of β-catenin. It is also confirmed that Siah-1 induces ubiquitination of the membrane bound β-catenin regardless of p53 activation [148]. In addition, sarcolemma associated β-catenin is ubiquitinated by Ozz-E3 ligase [149], and β-catenin in the nucleus is ubiquitinated by Jade-1 to induce degradation [150]. Contrarily, ubiquitination of EDD, Rad6B, and FANCL increases the stability of β-catenin. EDD attaches the K29- and K11-linked polyubiquitin chains and Rad6B attaches the K63-linked polyubiquitin chain [21].

### 6.2. Destruction Complex

The destruction complex that induces degradation of β-catenin is regulated by ubiquitination. Axin is ubiquitinated by four E3 ligases such as Siah-1, RNF146, Smurf1, and Smurf2. The ubiquitination of Siah-1 occurs by binding to the VxP motif of Axin and functions as a positive regulator of the Wnt signaling pathway. RNF146 induces degradation to promote K48-linked ubiquitination of PARylated Axin. Ubiquitination of Smurf1 occurs in K505 of Axin and results in its degradation, whereas ubiquitination of Smurf1 does not cause degradation of Axin but disturbs the interaction between Axin and LRP6, thereby functioning as a negative regulator of the Wnt signaling pathway [151].

Another destruction complex component, GSK3 is ubiquitinated at T1695 by FVXW7 and is associated with Toll-like receptor-mediated cytokine production [152]. Moreover, CK1 is ubiquitinated and degraded by CRL4 (Cullin-RING E3 ubiquitin ligase), which is a lenalidomide dependent process [153]. The E3 ligase of APC was recently revealed to be MKRN1 (RNF61), which induces ubiquitination and degradation to bind armadillo repeat domain of APC. Since downregulation of RFN61 inhibits proliferation, invasion, migration, and expression of Wnt target genes [154], it is assumed that upregulation of RNF61 may be related to cancer growth.

### 6.3. Dvl

Ubiquitination of Dvl is caused by the several E3 ligases composed of HECT-like ubiquitin ligases and RING-type ubiquitin ligase. HECT-like ubiquitin ligase NEDD4 promotes ubiquitination and degradation of Dvl-1 by the activated Rac Family Small GTPase 1 (Rac1) that is related with cell to cell junctions [155]. Another HECT-like ubiquitin ligase, NEDD4L promotes Lys 6, Lys 27, and Lys 29 linked polyubiquitination and degradation of Dvl-2 subsequent to stimulation of Wnt5a [156]. The HECT-like ubiquitin ligase ITCH also attaches ubiquitin and triggers degradation of phosphorylated Dvl-2 to bind PPXY motif or DEP domain [157]. Furthermore, the homolog HECT domain ubiquitin ligase EEL-1 (Huwe1) targets Dvl by polyubiquitination of the DIX domain and affects Dvl multimerization, but not degradation, of Dvl [158]. The KLHL12-Cullin-3 ubiquitin ligase (RING-type ubiquitin ligase) promotes ubiquitin-mediated degradation of Dvl-3 in the presence of Wnt protein, and diminishes the Wnt signaling pathway [155]. Recent studies report found that oligomerization of the pleckstrin homology domain-containing protein (PLEKHA4) represses polyubiquitination of Dvl by KLHL12-Cullin-3 ubiquitin ligase, and that knockout of *Drosophila* PLEKHA4 affects the PCP signaling pathway [159]. Other RING-type ubiquitin ligases such as MARCH2 and Malin induce ubiquitination of Dvl. Malin bring about K48 and K63 linked ubiquitination of Dvl-2 in Wnt signaling of Lafora disease [160], and MARCH2 bring about Dvl ubiquitination with Dapper1, and also plays an important role in vertebrate head development [161]. Regulating the stability of Dvl through ubiquitination is important as a mediator of Wnt signaling pathway, since Dvl acts on both the noncanonical and canonical pathways.

### 6.4. Wnt Receptors

Stability of Fz and LRP6 receptors is related to the accumulation of Wnt receptor and it makes Wnt receptors more sensitive in the presence of Wnt protein [32]. Removal of the Fz receptor at the cell surface is mediated by ubiquitination of RNF43/ZNRF3, a transmembrane RING-type E3 ligase. The extracellular cysteine-rich domain of Fz and ectodomain of RNF43/ZNRF3 are required in this process [162]. Ubiquitination of the Fz receptor by RNF43/ZNRF3 is regulated by binding of RSPO to the Rhodopsin G-protein coupled receptor family members such as LGR4 and LGR5. Binding of RSPO to LGR4/5 and RNF43/ZNRF3 through the Furin domain triggers auto ubiquitination and clearance of RNF43/ZNRF3 [163]. Therefore, the Wnt signaling pathway is positively induced by the increased stability of Wnt receptors [164]. Ubiquitination of LRP6 plays an important role in the folding process of LRP6. In the ER, ubiquitination of LPR6 K1043 is retained without degradation. Only LRP6, which has been detached ubiquitin by a DUB, USP19, is able to freely move through the ER to the cell membrane [165].

### 6.5. TCF/LEF

In the intranuclear process directly associated with the expression of Wnt target genes, the TCF family competitively binds with β-catenin and transducin-like enhancer (TLE) family [166]. In the absence of Wnt protein, TCF functions as a negative regulator to bind with the TLE family due to the ubiquitination-mediated degradation of cytoplasmic β-catenin [2]. However, in the presence of Wnt protein, the accumulated β-catenin is translocated into the nucleus, and binding between TCF and β-catenin promotes the transcription of Wnt target genes. The TCF/LEF1 are targeted by a RING-type E3 ligase, Neurodap1 (Pja2), which triggers ubiquitination and downregulates the protein levels of TCF/LEF1 LEF1 [167,168]. This process is associated with self-renewal and differentiation of embryonic stem cells [167]. EDD, also known as E3 ligase of β-catenin, promotes ubiquitin-mediated degradation of the TCF/LEF family [21]. Nemo-like kinase-associated ring finger protein (NARF) attaches the ubiquitin to TCF/LEF with the E2 ubiquitin ligase E2-25K, and represses formation of axis in *Xenopus* embryo [169].

**Figure 4:**
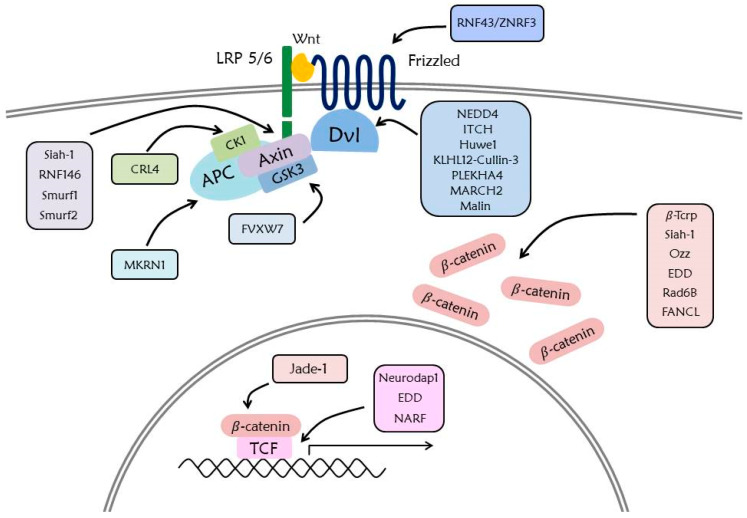
Ubiquitination in the Wnt signaling pathway. Numerous E3 ligases regulate the stability or activity of Wnt signaling factors inducing ubiquitination. Most proteins in the Wnt signaling pathway are regulated by ubiquitination, and many E3 ligases that regulate β-catenin, Dvl, and Axin are well known. The ubiquitination of Wnt signaling pathway has an important role in protein folding and stability through proteasomal degradation.

It was found that the PTM by ubiquitination regulates the stability of the primary Wnt signaling pathway components described above. Diverse E3 ligases participate in the regulation of the Wnt signaling pathway [151] (Figure 4). It has also been suggested that diverse DUBs that detach ubiquitin by E3 ligase are associated with Wnt signaling factors.

## 7. Deubiquitination in Wnt Signaling Pathway

### 7.1. USP2a

USP2a promotes the nuclear accumulation and transcriptional activity of β-catenin, thereby increasing expression of the Wnt/β-catenin target gene. USP2a directly binds to β-catenin, and the ARM domain or the C-terminal domain of β-catenin is essential for the interaction with USP-2a. Reducing the expression of USP2a or suppressing it through small molecules causes β-catenin destabilization. This indicates that USP2a can act as a therapeutic target for the cancer-promoting protein β-catenin [170].

### 7.2. USP4

USP4 is a β-catenin specific DUB. USP4 regulates β-catenin and promotes β-catenin-regulated transcription. The C-terminal catalytic domain of USP4 is responsible for β-catenin binding and nuclear transport. The expression of USP4 and β-catenin was found to be enhanced in colon cancer tissues from patients. In addition, USP4 knockdown in a colon cancer cell line resulted in decreased invasion and migration. This indicates that USP4 is a positive regulator of Wnt/β-catenin signaling. Therefore, it is predicted that USP4 could be a potential target for anticancer drugs [171].

### 7.3. USP6

siRNA screening targeted at Wnt/β-catenin reporter activity was performed to identify DUBs related to Wnt/β-catenin signaling. It was determined that USP6 exerted maximum effect on Wnt/β-catenin [172,173]. Decreased USP6 expression resulted in decreased expression of the Wnt/β-catenin target gene. Increased expressions of USP6 and USP6 catalytic mutant at various doses resulted in dose-dependent increase of Wnt/β-catenin in USP6 expressed cells, but no significant difference was observed in the USP6 catalytic mutant. Further investigation on the mechanism of action of USP6 in Wnt/β-catenin signaling determined that USP6 affected upstream of the β-catenin destruction complex. USP6 overexpression resulted in an increased level of Fz, and ubiquitination of Fz decreased with increased USP6 expression [174]. These results indicate that USP6 regulates Wnt/β-catenin signaling by deubiquitination of Fz [173].

### 7.4. USP6NL

USP6NL, a homolog of USP6, has a domain that encodes a deubiquitination enzyme [175]. The expression level of USP6NL was higher in tumor tissues of colorectal cancer (CRC) patients. Knockdown of *USP6NL* suppressed cell proliferation, and altered the expressions of P27, Cyclin D1, and c-Myc; target genes of β-catenin were involved in the transition from G0/G1 to S phase [176,177]. USP6NL interacts with β-catenin and reduces ubiquitination of β-catenin. This indicates that USP6NL activates the Wnt/β-catenin signaling while deubiquitination of β-catenin increases cell proliferation in CRC and increases cell cycle progression. Thus, *USP6NL* acts as an oncogene for CRC [177].

### 7.5. USP7

USP7 represses Wnt/β-catenin signaling through stabilization of Axin, which is a part of the destruction complex that inhibits β-catenin. Increased Axin expression decreases the level of activated β-catenin, subsequently suppressing Wnt/β-catenin signaling. Axin is degraded through ubiquitination. Deubiquitination of Axin mediated by USP7 prevents Axin from degrading and providing stability. The TRAF domain of USP7 and amino acids 32–245 of Axin bind directly. Stabilization of Axin through USP7 reduces the expression of β-catenin, as well as target genes of β-catenin. Hence, this process affects Wnt/β-catenin signaling [178]. Conversely, USP7 promotes deubiquitination of β-catenin with RNF220, which is a RING domain E3 ligase, and upregulates the Wnt signaling pathway [179]. Therefore, USP7 functions as both positive and negative regulator for the Wnt signaling pathway.

### 7.6. USP9X

A previous study reported the involvement of Wnt/β-catenin signaling and BCL9 in cancer [180]. Amino acids 1101 to 1553 of USP9X are the sites that interact with BCL9. USP9X removes K63 linked polyubiquitin on K212 of BCL9. The USP9X-mediated BCL9 deubiquitination promotes formation of the β-catenin-BCL9-PYGO complex, increasing the transcriptional activity of the Wnt/β-catenin target genes. Therefore, USP9X has an important role in regulating the Wnt/β-catenin signaling. Additionally, the deubiquitination of BCL9 by USP9X increases proliferation and invasion of breast cancer cells. This indicates that USP9X is involved in Wnt/β-catenin signaling and breast carcinogenesis [181].

### 7.7. USP14

Dvl plays an important role in Wnt signaling [39]. Dvl is ubiquitinated at the C-terminus. Mutations in the ubiquitin domain cause polyubiquitin, which is primarily K63-linked. USP14 functions as a DUB of Dvl and promotes deubiquitination of Dvl. Inhibition of USP14 increases Dvl polyubiquitination and significantly impairs downstream Wnt signaling. This indicates that USP14 functions as a modulator of the Wnt signaling pathway. In colon cancer, the carcinogenic role of USP14 is demonstrated throughan increased level of Wnt/β-catenin signaling [182].

### 7.8. USP15

COP9 signalosome (CSN) is involved in Wnt/β-catenin signaling by regulating β-catenin and APC [183,184]. CSN interacts with Cullin-RING ubiquitin ligases (CRLs) and the β-catenin destruction complex. CSN promotes the degradation of β-catenin and stabilizes APC. The stabilization of APC proceeds through CSN-associated USP15, which is confirmed by observing increased degradation of APC with deceased expression of USP15. Thus, USP15 is involved in the stability of CSN, CRL, and the β-catenin destruction complex through stabilization of APC, indicating that it affects Wnt/β-catenin signaling [184].

### 7.9. USP25

Proteins that bind to USP25 were discovered through mass spectrometry, and one of the proteins identified was tankyrase. Tankyrase is known to affect Wnt/β-catenin signaling by degrading Axin and thereby stabilizing β-catenin [185]. Tankyrase and USP25 interact with four ankyrin repeat clusters (ARCs) of the N-terminal of tankyrase and the C-terminal of USP25. Levels of tankyrase expression are directly proportional to the expression of USP25. Moreover, tankyrase is stabilized by USP25. It is also confirmed that increased levels of USP25 expression result in decreased Axin1 and increased β-catenin expression. This suggests that USP25 acts as a DUB of tankyrase to stabilize tankyrase and induce a positive process of Wnt/β-catenin signaling [186].

### 7.10. USP34

In the LC-MS/MS analysis of AXIN1 and AXIN2, USP34 was found to be associated and have an endogenous interaction with AXIN1 [187,188]. USP34 reduced the ubiquitination of AXIN1, but the catalytic mutant of USP34 does not reduce the ubiquitination of AXIN1. Also, decreased USP34 expression resulted in decreased expression of AXIN1, and increased expression of β-catenin. This indicates that USP34 suppresses Wnt/β-catenin signaling, due to degradation of β-catenin resulting from increased stability of AXIN1 [188].

### 7.11. USP44

*USP44* mRNA expression was decreased in colorectal cancer (CRC) [189]. Increased expression of USP44 results in increased expression of Axin1, and decreased expressions of β-catenin, c-Myc, and Cyclin D1. This indicates that increasing the expression level of USP44 inhibits Wnt/β-catenin signaling. USP44 does not affect the expression of *Axin1* at the mRNA level, but affects the protein level, and interacts with Axin1. The ubiquitination of Axin1 decreases with increased expression of USP44. It was also confirmed that when USP44 increased, it promoted apoptosis of CRC cells. This indicates that USP44 deubiquitination of Axin1 increases β-catenin degradation, inhibits Wnt/β-catenin signaling, and affects CRC [190].

### 7.12. USP47

β-catenin is known to be ubiquitinated by β-Trcp [23]. USP47 directly interacts with β-catenin and affects Wnt/β-catenin signaling by deubiquitination of β-catenin. Decreasing the expression of USP47 increases the ubiquitination of β-catenin. Differences between USP47 and a catalytic mutant USP47 were confirmed, and it was observed that ubiquitination of β-catenin increases with a mutant form [153,191]. The results indicate that USP47 induces a positive process in Wnt/β-catenin signaling while increasing the stability of β-catenin [8,153].

### 7.13. USP51

USP51 expression was found to be high in pancreatic cells from cancer patients. Knockdown of USP51 decreases the cell proliferation and halts the cell cycle at G0/G1 phase. Increased expression level of USP51 resulted in increased expression of Cyclin D1, a target gene of β-catenin. USP51 interacts with β-catenin and reduces ubiquitination of β-catenin. This indicates that USP51 acts as an oncogene that increases the expression level of β-catenin, activates Wnt/β-catenin signaling, and increases cell proliferation in PC by deubiquitination of β-catenin [192].

### 7.14. CYLD

Dvl is the key component in Wnt signaling. The presence of Dvl prevents the constant destruction of β-catenin [39]. Dvl was found to be polyubiquitinated mainly through ubiquitin K63, and the DIX domain of Dvl is mainly ubiquitinated. The DUB of Dvl found through DUB screening is CYLD. Decreased CYLD expression resulted in increased ubiquitination of Dvl and increased β-catenin expression. Moreover, the expression of Axin2, one of the target genes of the Wnt signal, was also increased. This suggests that stabilization of Dvl through CYLD affects Wnt/β-catenin signaling while increasing β-catenin [193].

### 7.15. UCH37

TCF/LEF family proteins play an important role in Wnt/β-catenin signaling [67]. UCH37 interacts with the HMG domain of TCF7 and C-terminal amino acids 240–365 [194,195]. The increased expression of UCH37 decreases the polyubiquitin of TCF7. K63- and K48-linked polyubiquitin chains were removed from TCF7protein when UCH37 expression was increased. Changes in the expression level of UCH37 did not affect the expression level of TCF7 protein. This suggests that UCH37 does not affect TCF7 protein stability. However, deubiquitination of TCF7 by UCH37 is involved in TCF7 binding to the promoters of c-Myc, and Cyclin D1 [194,196]. This suggests that UCH37 affects Wnt/β-catenin signaling [194].

**Figure 5:**
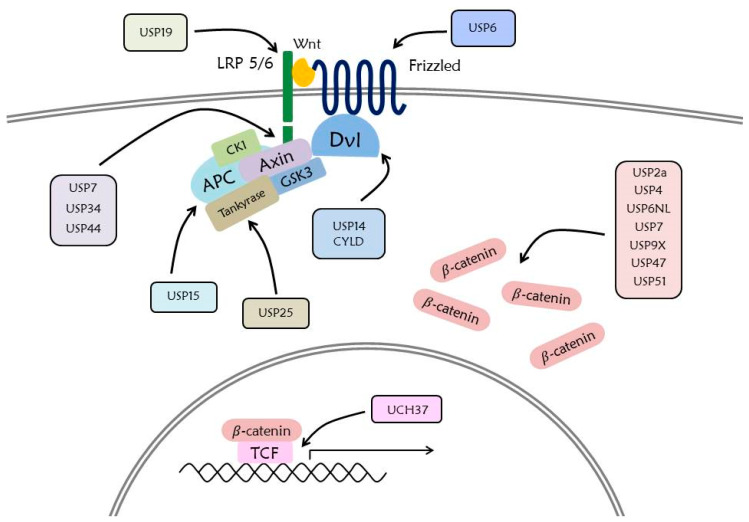
Deubiquitination in the Wnt signaling pathway. Deubiquitination of the Wnt signaling pathway by DUBs mainly functions to increase the stability of proteins, detaching ubiquitin from the target protein. DUBs associated with Wnt signaling pathway are mostly members of the USP family.

These numerous DUBs are associated with regulation of the Wnt signaling pathway (Figure 5), which is involved in cell proliferation, migration, and EMT. This suggests that DUB inhibitors can be applied for therapeutic strategies in different contexts.

## 8. DUB Inhibitors and Small Molecules

Regulating stability and activity of target proteins through ubiquitination and deubiquitination is associated with diverse cellular pathways and diseases. DUBs control homeostasis of target proteins by preventing degradation by UPS and by increasing stability. Thus, inadequate expression of DUB is also an implication in cancers as well as other diseases. Hence, numerous inhibitors have been identified for DUBs (Table 2) [197]. Cancers arising due to dysregulation of the Wnt signaling pathway can be regulated by numerous DUBs targeting the stability of Wnt signaling components. Through specific inhibitors, modulation of DUBs that target Wnt signaling components could be a potential anticancer therapy in diverse human cancers caused by dysregulation of Wnt signaling pathway.

Inhibitors for USP7 have been well studied among the DUB families, since USP7 regulates cancer related proteins such as MDM2, p53, PTEN, and FOXO [198]. USP7 is known to be highly expressed in colorectal cancer cells and tissues and is associated with poor prognosis. One of the USP7 inhibitors, P5091 represses the Wnt signaling pathway by enhancing ubiquitin-mediated degradation of β-catenin in CRC cells as well as in in vivo models [199]. It was confirmed that the attenuation of USP7 by another USP7 inhibitor (P20077) inhibits the APC mutated CRC tumor growth in a xenograft model [200]. A recent study also demonstrated that sesquiterpene lactone parthenolide (PTL) downregulates the Wnt signaling pathway by lowering the stability of β-catenin through inhibition of USP7 activity [201].

Furthermore, the USP14 inhibitor b-AP15 is known to induce ER stress and represses liver cancer cell growth through regulation of the Wnt signaling pathway [202]. Effects of USP14 inhibitor IU1 confirmed that inhibition of USP14 by IU1 increases the K4-linked polyubiquitination of Dvl [182].

In addition to USP7 and USP14, a number of inhibitors for DUB involved in the deubiquitination of Wnt signaling components have been identified and developed [203]. However, up to date, there exist no DUB inhibitors that can be used for clinical purpose, whereas Wnt signaling inhibitors (such as WNT974, a PORCN inhibitor) are used in metastatic CRC and diverse human cancers [65]. To indicate the potential value of the DUB inhibitors, we decided to add some information about. VLX1570, known as an inhibitor of USP14 and UCHL5, is in the middle of a clinical trial (Phases 1 and 2) to confirm its stability and efficiency for the multiple myeloma (NCT02372240). VLX1570 is known to induce apoptosis of myeloma by targeting USP4 and UCHL5 [204]. Clinical studies of other DUB inhibitors including VLX1570 will play an important role in anticancer therapy. Thus, clinical trials of DUB inhibitors are an important process for cancer therapy. Also, investigating DUB inhibition activity of anticancer drugs or potential anticancer drugs will also be important for cancer treatment approaches.

## 9. Conclusions

Over the past few decades, numerous researches have focused on the association of Wnt signaling pathway and human cancers. It has been found that the modification of stability or activity of each component in the Wnt signaling pathway through gene mutation or PTM causes and inhibits diverse human cancers. Thus, a mechanism controlling Wnt signaling has an important implication for targeting cancers by dysregulation of Wnt signaling factors. Ubiquitination and deubiquitination are usually known to regulate protein levels by ubiquitin mediated proteasomal degradation. Diverse E3 ligases and DUBs are associated with Wnt signaling factors, resulting in upregulation or downregulation of Wnt target genes such as c-Myc and Cyclin D1, which are related to tumor growth. Many researches have determined the E3 ligase and DUB target Wnt signaling factors, as well as sites of target proteins which undergo ubiquitination and deubiquitination. However, many aspects remain unclear in the ubiquitination and deubiquitination mechanism for Wnt signaling factors, and future studies require a full understanding of the adjustment of Wnt signaling through ubiquitination and deubiquitination. The balance between these mechanisms will be significant for modulating the Wnt signaling pathway and deriving potent therapeutic strategies in different contexts.

## Figures and Tables

**Table 1:** Wnt signaling factors in various cancers.

Factors	Cancer Type	Feature	References
APC	Colorectal cancer	Truncated mutation	[46]
	Gastric cancer	APC promoter methylation	[73]
		Genetic mutations	[74]
	FAP	Mostly point mutation	[75]
	Pancreatic cancer	Genetic mutations	[76]
		Hypermethylation	[77]
	Liver cancer	APC promoter methylation	[78]
	Thyroid cancer	Genetic mutations	[79]
	Breast and lung cancers	Hypermethylation	[77]
	Brain cancer	Genetic mutations	[49]
Axin1/2	Adrenal cancer	Deletion (Axin2)	[80]
	Breast cancer	Low expression (Axin2)	[46]
	Colorectal cancer	Hypermethylation (Axin2)	[77]
		Inactivating mutations (Axin2)	[77]
	Skin and liver cancers	Inactivating mutations (Axin1)	[77]
	Ovarian cancer	Nonsense mutation (Axin1)Frame shift mutation (Axin2)	[81]
	Brain cancer	Genetic mutations (Axin1)	[49]
	Bladder cancer	Polymorphisms (Axin1)	[52]
	Lung cancer	Polymorphisms (Axin2)	[53]
GSK3	Colon, liver, ovarian, and pancreatic cancers	High expression	[54]
	Gastric cancer	Differential phosphorylation residues	[82]
CK1	Bladder, brain, breast, colorectal, kidney, lung, ovarian, pancreatic, prostate, and hematopoietic cancers	High expression	[55]
LRP5/6	Skin cancer	Inactivating mutation	[77]
	Colorectal, liver, breast, and pancreatic cancers	High expression	[36]
	Lung, bladder, colorectal cancers	polymorphism	[36]
Fz	Nerve, liver, lung, endometrial, colorectal, leukemia, prostate, cervical, esophageal, glioma, bone, synovial sarcoma	High expression	[59]
	Salivary gland cancer	Low expression	[59]
Dvl	Lung, prostate, breast, cervical, and gliomas cancers	High expression	[62]
RNF43/ZNRF3	Pancreatic, adrenal cancers	Inactivating mutations (ZNRF3)	[32]
	Ovarian, stomach, pancreatic, colorectal, endometrial, and liver (Biliary tract) cancers	Inactivating mutations (RNF43)	[32]
DKK1	Colorectal, breast, gastric, and ovarian cancers	DNA methylation	[32]
	Bile duct, bone, liver, bladder, breast, pancreatic, skin, prostate, esophageal, and laryngeal cancers	High expression	[83]
	Thyroid, colorectal, cervical, and lung cancers	Low expression	[83]
RSPO	Large intestine, lung, esophagus, stomach, ovary, and breast cancers	Chromosome rearrangement	[32]
CTNNB1	Liver, endometrium, adrenal, large intestine, stomach, skin, and pancreatic cancers	Mainly missense mutation	[32,77]
	Ovarian cancer	Activating mutations	[81]
β-Trcp	Lung cancer	Negatively regulating F-box protein	[84]
		Negatively regulating Mxi1	[71]
	Gastric cancer	Genetic mutations	[85]
TCF/LEF	Liver cancer	Expression of TCF isoform	[86]
	Colon, and intestine cancers	Expression of TCF4	[67]
PORCN	Esophageal, ovarian, uterus, lung, and cervical cancers	Genetic mutations	[65]

**Table 2:** Identified DUB inhibitors that have influence on signaling components in the Wnt signaling pathway.

DUB	Target Protein in Wnt Signaling	DUB Inhibitors	References
USP2a	β-catenin	ML364	[205]
USP4	β-catenin	Vialinin A, Neutral red	[206,207]
USP7	Axin, β-catenin	P5091, HBX-41108, P20077	[198]
USP9X	BCL9 (β-catenin-BCL9-PYGO complex)	Degrasyn	[208]
USP14	Dvl	IU1, b-AP15	[209,210]
USP15	β-catenin, APC	UbV	[211]
USP19	LPR6	I-217	[203]
USP25	Tankyrase	AZ1	[203]
USP47	β-catenin	P22077	[203]
CYLD	Dvl	Subquinocin	[212]
